# Effects of Electroacupuncture on Sleep via the Dopamine System of the HPA Axis in Rats after Cage Change

**DOI:** 10.1155/2021/5527060

**Published:** 2021-07-01

**Authors:** Chen Xie, Jing Wang, Na Zhao, Wenjia Yang, Xiaolin Gao, Zhen Liu, Xinyu Chen, Chaojun Fang, Cong Fu, Yunfei Chen, Xintong Yu

**Affiliations:** ^1^Yueyang Hospital of Integrated Traditional Chinese and Western Medicine, Shanghai University of Traditional Chinese Medicine, Shanghai 200437, China; ^2^Shanghai Research Institute of Acupuncture and Meridian, Shanghai University of Traditional Chinese Medicine, Shanghai 200030, China; ^3^Beijing University of Chinese Medicine Dongfang College, Langfang 065001, China

## Abstract

**Background:**

Insomnia is often related to stressful events. The hypothalamus-pituitary-adrenal (HPA) axis is related to stress, and dopamine (DA) and DA receptors are involved in the regulation of HPA axis. Electroacupuncture (EA) can improve sleep in individuals with insomnia, but the mechanism is unclear. We demonstrated that EA can improve sleep in rats after cage change through DA and the DA receptors in the HPA axis.

**Methods:**

A rat model of insomnia was established by cage change to a dirty cage. The rats in treatment groups were intervened by EA and D1R (or D2R) antagonists. Electroencephalography (EEG) and electromyogram (EMG) were recorded to compare the changes in sleep. The DA, corticotropin-releasing hormone (CRH), adrenocorticotropic hormone (ACTH), and cortisol (CORT) levels in the plasma and hypothalamus were measured by ELISAs, and the D1R and D2R levels were measured by RT-PCR and immunohistochemistry.

**Results:**

The dirty group showed a significant increase in the amount of wakefulness and decrease in the amount of NREM sleep, with decreased numbers of long NREM sleep bouts and REM sleep bouts and increased mean duration of wakefulness during the light period. EA and D1R (or D2R) antagonists intervention could improve sleep disturbance by decreasing wakefulness in the light period after cage change, EA and D1R (or D2R) antagonists could increase the hypothalamus DA, CRH, ACTH, CORT level, and the D1R and D2R mRNA levels in the HPA axis, and the effect of EA plus D1R (or D2R) antagonist was not superior to that of EA or D1R (or D2R) antagonists alone.

**Conclusions:**

EA can improve the sleep of rats after cage change, and the mechanism may be related to the regulation of DA and D1R or D2R in the HPA axis.

## 1. Introduction

Insomnia is a highly prevalent disease that can occur at all ages, and many social or societal stressors are associated with insomnia [1]. More than 20% of the population suffers from insomnia-related disorders based on the DSM-IV, DSM-5, or ICD-10, and the prevalence of insomnia is 33% to 50% in adults [2–4]. In addition to the increased healthcare costs, insomnia negatively affects patients' quality of life (QOL) [5, 6]. Insomnia can cause daytime dysfunction, affect normal study and work, and eventually lead to a decline in the quality of life and a series of physiological and psychological diseases [7]. For example, people with insomnia are 7 times more likely to suffer from depression than healthy people [8], and insomnia will increase the risk of anxiety [9], cardiovascular diseases [10, 11], and suicide [12]. Moreover, insomnia is associated with an increased risk for all-cause and cardiopulmonary mortality and is associated with a steeper increase in inflammation [13]. Insomnia is also linked to higher rates of traffic accidents [14].

Insomnia may be the result of the combined effect of sleep mechanisms and stress-response-related gene-environment effects on brain plasticity [15]. The pathophysiological mechanism of insomnia is excessive awakening during the sleep-wake cycle [16]. Numerous clinical and experimental studies have shown that stress is associated with poor sleep quality [17–19].

The occurrence of stress is related to the hypothalamic-pituitary-adrenal (HPA) axis, which induces an increase in glucocorticoids when strongly activated. The HPA axis plays important roles in modulating sleep, and increased HPA activity promotes sleep fragmentation; however, this same sleep fragmentation increases cortisol levels [20–23]. Stress can activate the dopamine (DA) system, and the activity of dopaminergic neurons in the ventral tegmental area (VTA) of the rat midbrain, which regulates ethologically relevant sleep-wake behaviors, is increased under stress (social defeat) [24–26]. Dopamine is related to sleep and wakefulness and acts as a wakefulness-promoting agent [27]. DA, acting through both D1 and D2 receptors, exerts a stimulatory effect on the excitability of the HPA axis induced by stress, and D1R and D2R antagonists can reduce the activity of the HPA axis under stress [28], which suggests that dopamine and its receptors in the HPA axis may be involved in the regulation of sleep-wake cycles in insomnia.

Acupuncture could decrease the perception of stress and modulate the stress response [29, 30]. As a form of acupuncture, electroacupuncture (EA) reinforces the function of acupuncture by continuous stimulation of acupoints with a small electric current. Accumulating evidence indicates that EA could attenuate the stress response [31–33] and regulate the HPA axis [34, 35] and DA in the brain [36].

Based on the abovementioned data, combined with our previous work implicating dopamine receptors in the HPA axis in the sleep-wake regulation of EA preconditioning in a rat model of insomnia due to cage change [37], EA may improve the sleep-wake cycle in rats under the cage change model of insomnia by changing the dopamine and dopamine receptor levels in the HPA axis. To test this hypothesis, we examined the effects of EA and dopamine receptor antagonists on sleep-wake patterns and the expression of dopamine and its receptors in the HPA axis in insomniac rats under a cage change model of insomnia.

## 2. Materials and Methods

**Figure 1:**
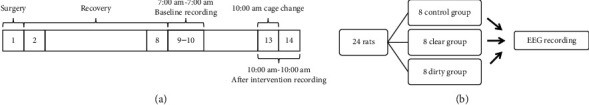
Timeline and group assignment. Rats were implanted with EEG and electromyogram (EMG) electrodes (day 1) and then housed in special cages and habituated to the recording cable for 5 days and recorded for 24 h as baseline EEG beginning at 7:00 am (days 9-10), subsequently exposed to intervention (same cage, clear cage, and dirty cage already occupied by another rat for five days) and recorded for 24 h beginning at 10:00 am (days 13-14) as EEG after intervention (a). Twenty-four rats were used in total, with eight in each group (b).

An overview of the first study design is shown in Figure 1(a); group assignment and sample sizes are shown in Figure 1(b). First, twenty-four rats were implanted with clectroencephalography (EEG) and electromyogram (EMG) electrodes. After a 7-day recovery period, rats were housed in special cages and habituated to the recording cable for 5 days and recorded for 24 h as baseline EEG beginning at 7:00 am (days 9-10). Then, rats were exposed to cage change as an acute insomnia model at 10:00 am (day 13) and recorded for 24 h beginning at 10:00 am (days 13-14) as EEG after intervention.

**Figure 2:**
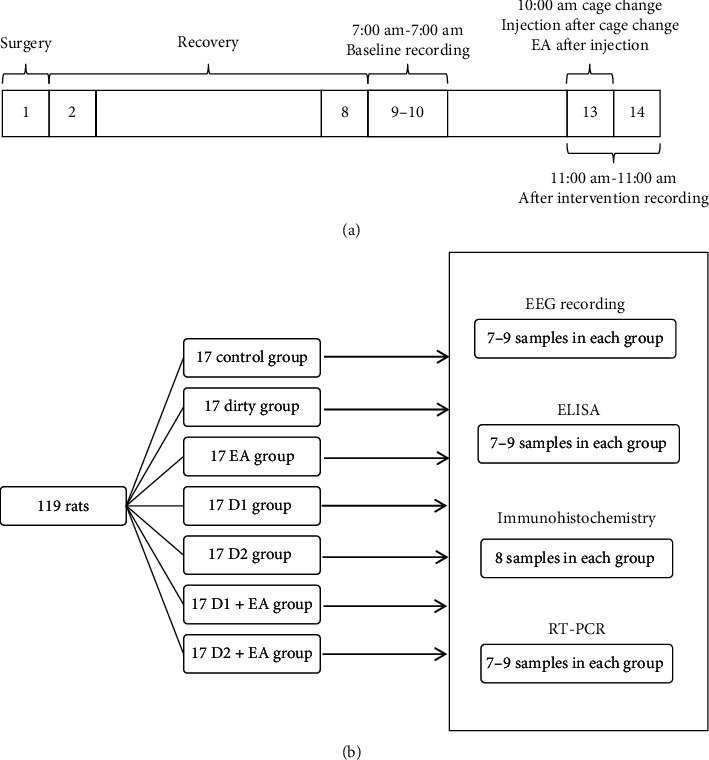
Timeline and group assignment. Rats were implanted with EEG and electromyogram (EMG) electrodes (day 1) and then housed in special cages and habituated to the recording cable for 5 days and recorded for 24 h as baseline EEG beginning at 7:00 am (days 9-10), subsequently exposed to cage change and intervention (the control group was injected with saline at 10:00 am, the dirty group was transferred to a dirty cage already occupied by another rat for five days at 10:00 am and then injected with saline, the EA group was treated with EA immediately after the cage change and injection, the D1 group was injected with SCH2 3390 after cage change, the D2 group was injected with raclopride after cage change, the D1 + EA group was treated with EA immediately after cage change and SCH2 3390 injection, and the D2 + EA group was treated with EA immediately after cage change and raclopride injection) and recorded for 24 h beginning at 11:00 am (days 13-14) as EEG after intervention (a). One hundred and nineteen rats were used in total, with seventeen in each group, and Elisa, RT-PCR, immunohistochemistry, and EEG analyses included 7–9 samples (b).

An overview of the second study design is shown in Figure 2(a); group assignment and sample sizes are shown in Figure 2(b). First, sixty-three rats were implanted with electroencephalography (EEG) and electromyogram (EMG) electrodes. After a 7-day recovery period, rats were housed in special cages and habituated to the recording cable for 5 days and recorded for 24 h as baseline EEG beginning at 7:00 am (days 9-10). Then, rats were exposed to cage change as an acute insomnia model at 10:00 am (day 13) and subsequently given injection and (or) EA as intervention and recorded for 24 h beginning at 11:00 am (days 13-14) as EEG after intervention. Fifty-six rats were not implanted with EEG and EMG electrodes; they were housed in special cages for 5 days and then given intervention after exposed to cage change at 10:00 am (6^th^ day). Their blood, hypothalamus, pituitary, and adrenal glands were sampled. DA, corticotropin-releasing hormone (CRH), adrenocorticotropic hormone (ACTH), and cortisol (CORT) concentrations in the blood and hypothalamus were analyzed using ELISA, mRNA levels of D1R and D2R in the hypothalamus, pituitary, and adrenal glands were measured using RT-PCR, and the expression of D1R and D2R in the HPA hypothalamus, pituitary, and adrenal glands were measured using immunohistochemistry.

### 2.1. Animals

Male SPF Sprague Dawley rats (300–350 g, Shanghai SLAC Laboratory Animal Co., Ltd., SCXK (Hu) 2012-0002) were individually housed in polypropylene cages (special cage for sleep-wake biological analysis) in the experimental animal center (SYXK (Hu) 2013-0109), which was sound proof, with electrostatic shielding, under a constant temperature (24 ± 1)°C and humidity (60 ± 2)%. The light-dark cycle (illuminance≈100 lux) was 7:00 am–7:00 pm (12 h light/12 h dark). Food and water were available ad libitum. All experimental procedures were conducted in accordance with the National Institutes of Health Guidelines for the Care and Use of Animals and approved by the Yueyang Hospital of Integrated Traditional Chinese and Western Medicine, Shanghai University of Traditional Chinese Medicine Animal Care and Use Committee.

### 2.2. Insomnia Rat Model

At 10:00 am on the sixth day, each rat was transferred to a dirty cage occupied by another rat for five days to establish the cage change model of insomnia [7].

### 2.3. EA Treatment

EA was performed after the cage change. The rats were subjected to EA at Ganshu (BL 18) and Zu San Li (ST36) to depths of 3 and 6 mm with four stainless steel needles (0.16 mm × 13 mm; Suzhou Kangjian Medical Devices Co., Ltd, Suzhou, China), respectively. BL 18 is located in the upper back region, at the inferior border of the spinous of the 9th thoracic vertebra (T9), at the intercostal. ST36 is distal to the head of the tibia in a depression between the muscles of the cranial tibia and the long digital extensor. The needles were connected to an EA stimulator, and a constant electrical stimulus (2 Hz, 0.2 ms pulse width) was applied for 15 min. The intensity (6-7 mA) was adjusted until local muscle contractions were seen. During this process, the rats were free to move.

### 2.4. Surgery and EEG Recording

Under pentobarbital anesthesia (50 mg/kg, i.p.), the rats were chronically implanted with EEG and electromyogram (EMG) electrodes for polysomnographic recordings. The implant consisted of 2 stainless steel screws inserted through the skull into the cortex to collect EEG signals and 2 stainless steel wires into the dorsal neck muscles to collect EMG signals. All electrodes were attached to a microconnector and fixed to the skull with dental cement. After penicillin powder was sprinkled on the wound, the wound was stitched and injected intraperitoneally with antibiotics. After a 7-day recovery period, rats were housed in special cages and habituated to the recording cable for 5 days and recorded for 24 h as baseline EEG, beginning at 7:00 am, before the intervention. After the intervention, each animal was recorded for 24 h, beginning at 11:00 am.

The EEG and EMG signals of rats were amplified, filtered, and converted to digital analogs by a sleep bioanalysis system and then recorded by SleepSign software (Kissei Comtec, Nagano, Japan). At the end of the EEG/EMG recording, SleepSign analysis software was used to identify wake (W), nonrapid eye movement (NREMS), and rapid eye movement (REMS) with 4 s as an analysis unit according to the unified standard automatic scanning. After automatic scanning was completed, manual inspection was carried out if necessary.

### 2.5. Drugs

Raclopride (Sigma), a selective D2R antagonist, was dissolved in sterile saline at a dose of 2 mg/kg (i.p.). SCH2 3390 (Sigma), a selective D1R antagonist, was dissolved in sterile saline at a dose of 30 *μ*g/kg (i.p.)

### 2.6. ELISA

The blood samples were taken from the abdominal aorta and centrifuged at 3500 rpm for 5 min to extract the serum 24 hours after the intervention. The hypothalamus was taken and stored at −80°C immediately after blood was taken. Blood samples were removed, and the hypothalamus was homogenized for measurement according to the instructions.

### 2.7. Immunohistochemistry

Animals were deeply anesthetized with pentobarbital anesthesia (50 mg/kg, i.p.) and then perfused with 150 mL of saline followed by 200 mL of 4% paraformaldehyde through the heart. The adrenal gland, pituitary gland, and hypothalamus of the rats were removed, postfixed overnight in 4% paraformaldehyde, and then, equilibrated in 30% sucrose for a minimum of 48 hours. Finally, the adrenal gland, pituitary gland, and hypothalamus of the rats were stored in a refrigerator at 4°C. The tissues were sectioned into 4–7 *μ*m sections after paraffin embedding and repaired under high pressure using 0.01 M sodium citrate buffer solution for 15 minutes after being dewaxed. The tissues were washed several times in 0.02 M PBS after natural cooling, sealed with 3% H_2_O_2_, washed again in 0.02 M PBS, incubated with primary antibodies at 4°C overnight, and then, incubated with HRP-labeled broad-spectrum antibody for 20–30 minutes at room temperature. Finally, the tissues were stained with DAB at room temperature, counterstained with hematoxylin, dehydrated, cleared, and mounted with neutral balsam.

### 2.8. RT-PCR

Total RNA from the fresh hypothalamus, pituitary, and adrenal glands was extracted using the Ultraspec phenol kit (Biotecx, Houston, Texas, USA) according to the manufacturer's instructions. cDNA was subsequently synthesized from total RNA using the cDNA Synthesis Kit (Roche, Mannheim, Germany). Quantitative PCR (qPCR) for DR1 and DR2 was performed with a SYBR Green detection system on an ABI 7300 Real-time PCR System (Applied Biosystems, Foster City, California, USA) and expressed relative to glyceraldehyde-3-phosphate dehydrogenase (GAPDH) as an internal control. The cycling conditions were as follows: initial denaturation at 95°C for 10 min, 40 cycles of denaturation at 95°C for 15 s, annealing, and elongation at 60°C for 45 s. Sense and antisense primer sequences for qPCR were 5′-TGACTTCGGCTCTGAAATC-3′ and 5′-TGGACAGCAAACTCAACTC-3′ for DR1, 5′-CAGCAGAAGGAGAAGAAAG-3′ and 5′-GATGTTGAAGGTGGTGTAG-3′ for DR2, and 5′-GTCGGTGTGAACGGATTTG-3′ and 5′-TCCCATTCTCAGCCTTGAC-3′ for GAPDH, respectively. All PCR assays were performed in triplicate.

### 2.9. Statistical Analysis

All data are expressed as the mean ± SD. Total durations and structures of sleep-wake were compared among three groups (control, clean, and dirty groups) or the seven groups (control, dirty, EA, D1, D2, D1 + EA, and D2 + EA) by using one-way analysis of variance (ANOVA) followed by LSD (homogeneous variance) and Dunnett's T3 (uneven variance) tests. The durations and structures of sleep-wake were compared among the dirty, EA, D1 (or D2), and D1 (or D2) + EA groups by using analysis of variance of factorial design (2 × 2 factorial design). A value of *P* < 0.05 was considered statistically significant.

## 3. Results

### 3.1. Part 1

There was no significant difference in the durations and structures (numbers of sleep bouts and episodes, mean durations, and stage transition numbers) of the sleep-wake cycle among the three groups before cage change (*P* > 0.05).

#### 3.1.1. Effect of Cage Change on the Sleep-Wake Duration

**Figure 3:**
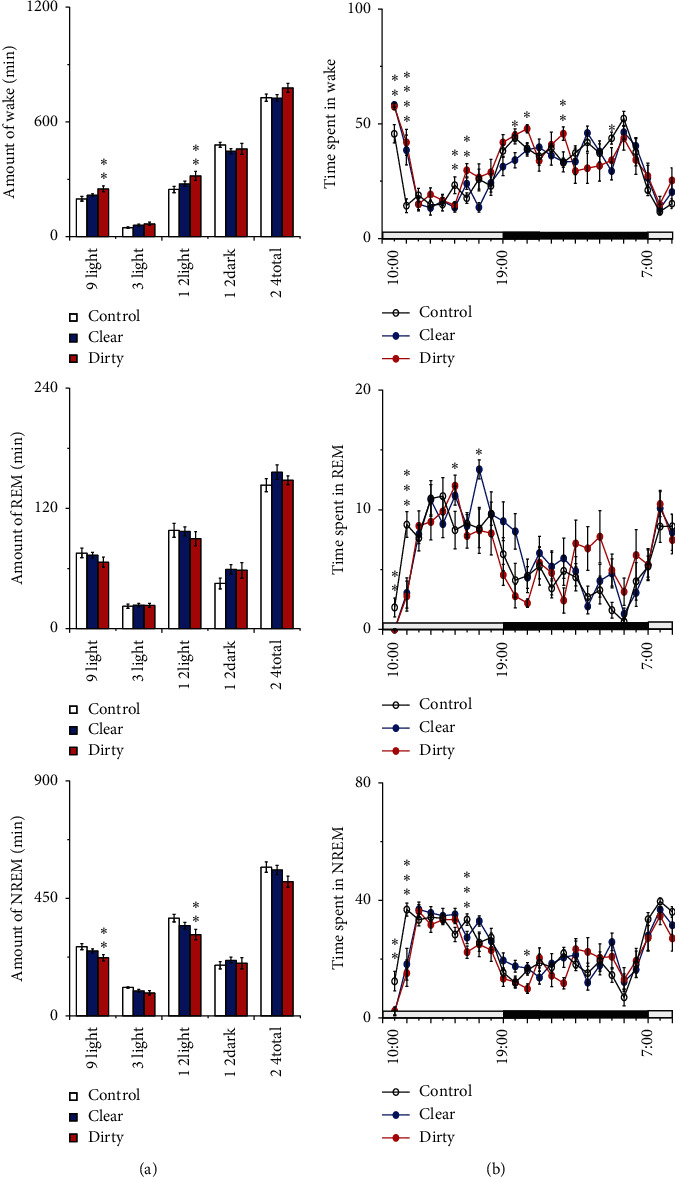
Sleep-wake profiles of the three groups after intervention. (a) Total time spent in wakefulness, NREM, and REM sleep during the 9 h light, 3 h light, 12 h light, 12 h dark, and 24 h total periods. Open bars, blue filled bars, and red filled bars show the profiles for the control group, clear group, and dirty group, respectively. (b) Time course changes in wakefulness, NREM, and REM sleep. Each circle represents the hourly mean amount of each stage. Open circles, blue filled circles, and red filled circles represent the control group, clear group, and dirty group, respectively. The horizontal open and filled bars on the axes indicate the light and dark periods, respectively. Values are the mean ± SEM (*n* = 8). Compared with the control group, ^*∗*^*P* < 0.05, ^*∗∗*^*P* < 0.01. Comparisons were performed using one-way ANOVA followed by Fisher's PLSD for each planned comparison.

Compared with that of the control group, the amount of wakefulness increased and NREM sleep decreased in the dirty group during the 9 h light period and 12 light period after intervention, and the difference was statistically significant (*P* < 0.05); compared with that of the control group, the amount of wakefulness and NREM sleep did not change in the clear group (the left side of Figure 3). Compared with that of the control group, the amount of wakefulness, NREM, and REM sleep changed in the clear and dirty group during the first, second, and seventh hours after cage change (*P* < 0.05) (the right side of Figure 3). These results indicate that rats placed to a dirty cage showed a significant increased amount of wakefulness and decreased amount of NREM sleep, especially in the first two hours and seventh hour after cage change, and rats placed to a clear cage still showed the amount of sleep-wake changed.

#### 3.1.2. Effect of Cage Change on the Numbers of Sleep Bouts and Episodes

**Figure 4:**
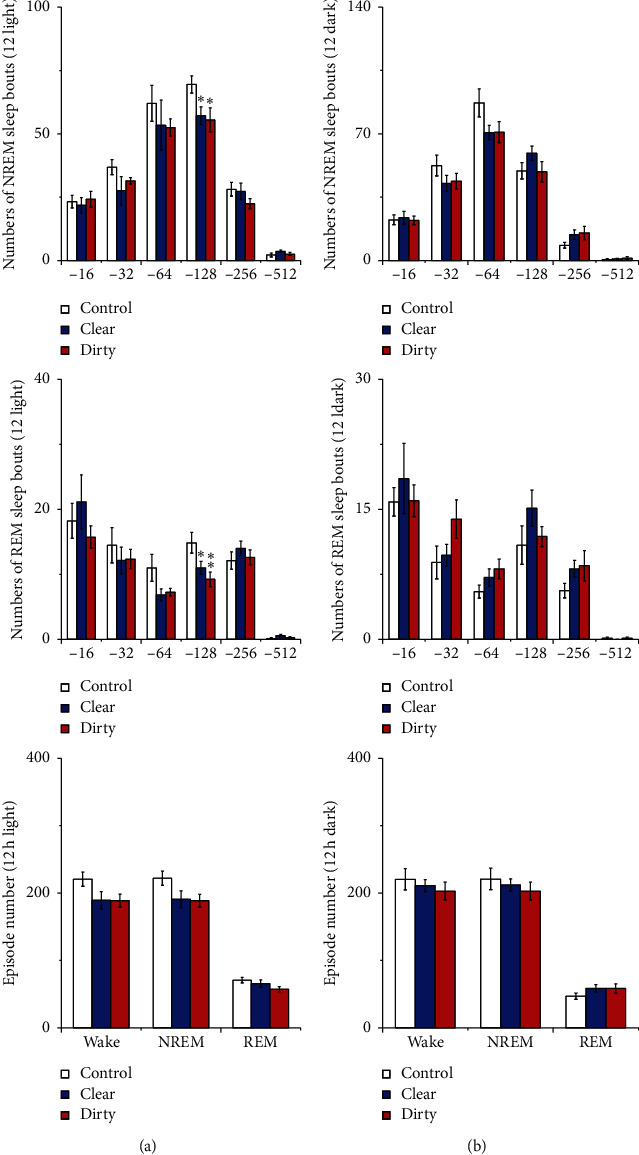
(a) Numbers of sleep bouts and (b) episode numbers. Open bars, blue filled bars, and red filled bars show the profiles for the control group, clear group, and dirty group, respectively. Values are the mean ± SEM (*n* = 8). Compared with the control group, ^*∗*^*P* < 0.05, ^*∗∗*^*P* < 0.01. Comparisons were performed using one-way ANOVA followed by Fisher's PLSD for each planned comparison.

Compared with that of the control group, the numbers of long NREM sleep bouts and REM sleep bouts during the light period decreased in the clear and dirty group (*P* < 0.05). See Figure 4 for details. These results indicate that rats placed to a dirty or clear cage had trouble in maintaining long NREM sleep bouts and REM sleep bouts during the light period.

#### 3.1.3. Effect of Cage Change on the Mean Durations of Wakefulness, NREM, and REM

**Figure 5:**
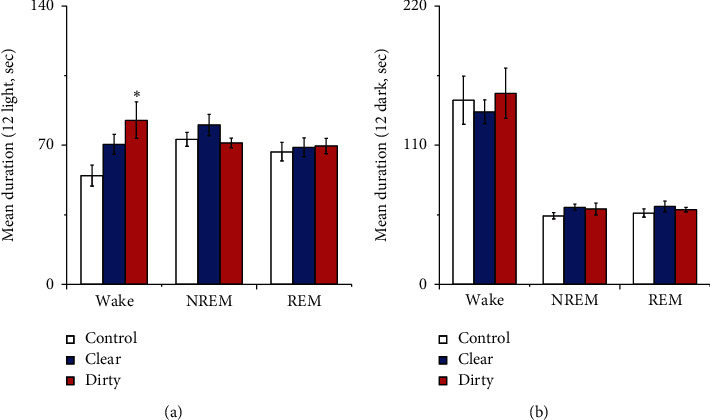
(a) Mean duration during the 12 h light period; (b) mean duration during the 12 h dark period. Open bars, blue filled bars, and red filled bars show the profiles for the control group, clear group, and dirty group, respectively. Compared with the control group, ^*∗*^*P* < 0.05, ^*∗∗*^*P* < 0.01. Comparisons were performed using one-way ANOVA followed by Fisher's PLSD for each planned comparison.

Compared with that of the control group, the mean duration of wakefulness during the light period increased in the dirty group (*P* < 0.05). See Figure 5 for details.

#### 3.1.4. Effect of Cage Change on the Stage Transition Numbers from Wakefulness, NREM, and REM

There was no significant difference in the stage transition numbers from wakefulness, NREM, and REM among the three groups after cage change (*P* > 0.05). These results indicate that rats after cage change did not show change in the stage transition.

### 3.2. Part 2

There was no significant difference in the durations and structures of the sleep-wake cycle among the seven groups before cage change (*P* > 0.05).

#### 3.2.1. Effect of EA on the Sleep-Wake Duration in the Rats after Cage Change

**Figure 6:**
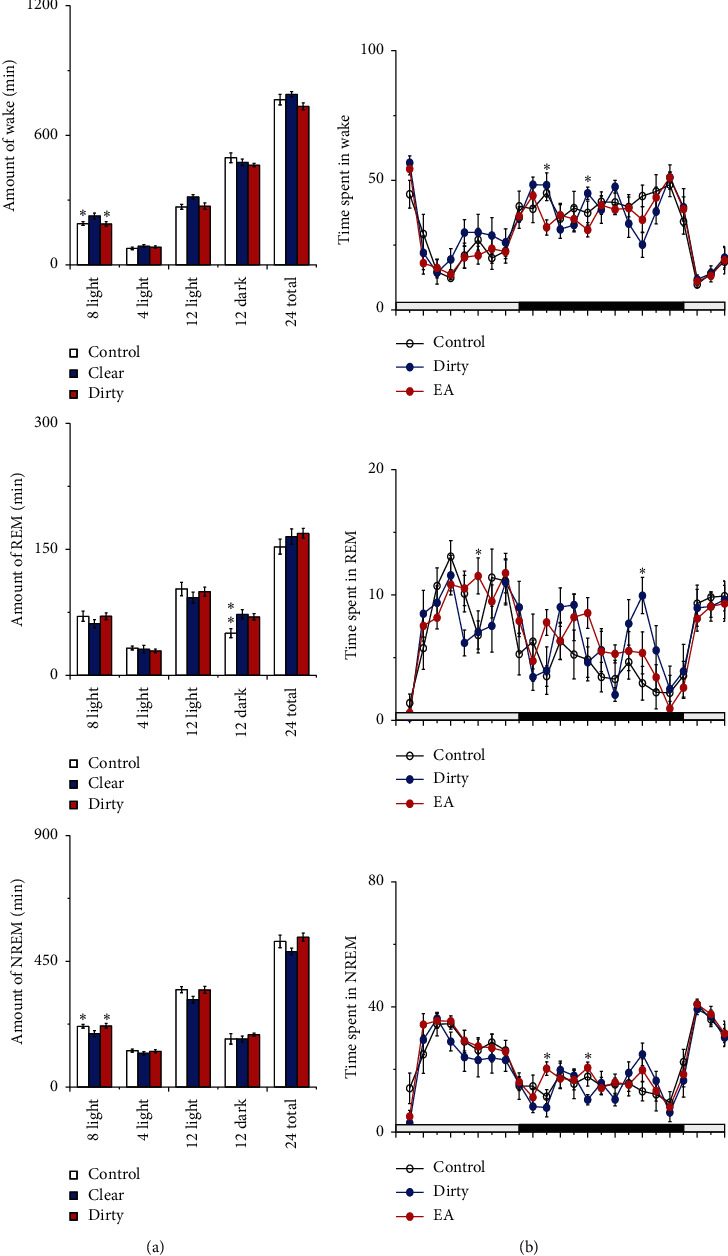
Sleep-wake profiles of the three groups after intervention. (a) Total time spent in wakefulness, NREM, and REM sleep during the 8 h light, 4 h light, 12 h light, 12 h dark, and 24 h total periods. Open bars, blue filled bars, and red filled bars show the profiles for the control group, dirty group, and EA group, respectively. (b) Time course changes in wakefulness, NREM, and REM sleep. Each circle represents the hourly mean amount of each stage. Open circles, blue filled circles, and red filled circles represent the control group, dirty group, and EA group, respectively. The horizontal open and filled bars on the axes indicate the light and dark periods, respectively. Values are the mean ± SEM (*n* = 7–9). Compared with the dirty group, ^*∗*^*P* < 0.05, ^*∗∗*^*P* < 0.01. Comparisons were performed using one-way ANOVA followed by Fisher's PLSD for each planned comparison.

*(1) The Sleep-Wake Durations among the Control, Dirty, and EA Groups*. Compared with that of the control group, the amount of wakefulness increased and NREM sleep decreased in the dirty group during the 8 h light period after intervention, and the difference was statistically significant (*P* < 0.05). Compared with that of the control group, the amount of REM sleep in the dirty group during the 12 h dark period increased (*P* < 0.05), and the difference was statistically significant (*P* < 0.05). Compared with that of the dirty group, the amount of wakefulness decreased and NREM sleep increased in the EA group during the 8 h light period after intervention, and the difference was statistically significant (*P* < 0.05). See Figure 6 for details. See Figure 6(b) for details of the amount of sleep-wake every hour. These results indicate that rats after cage change showed a significant increased amount of wakefulness and decreased in NREM sleep, and EA could decrease wakefulness and increase NREM sleep in rats after cage change.

*(2) The Sleep-Wake Durations among the Dirty, EA, D1, and D1 + EA Groups*. There was no interaction between EA and the D1R antagonist in the amounts of sleep-wake (*P* > 0.05), which indicated that the combined application of EA and the D1R antagonist did not have a better effect on the amounts of sleep-wake than a single application. See Table 1 for details.

**Figure 7:**
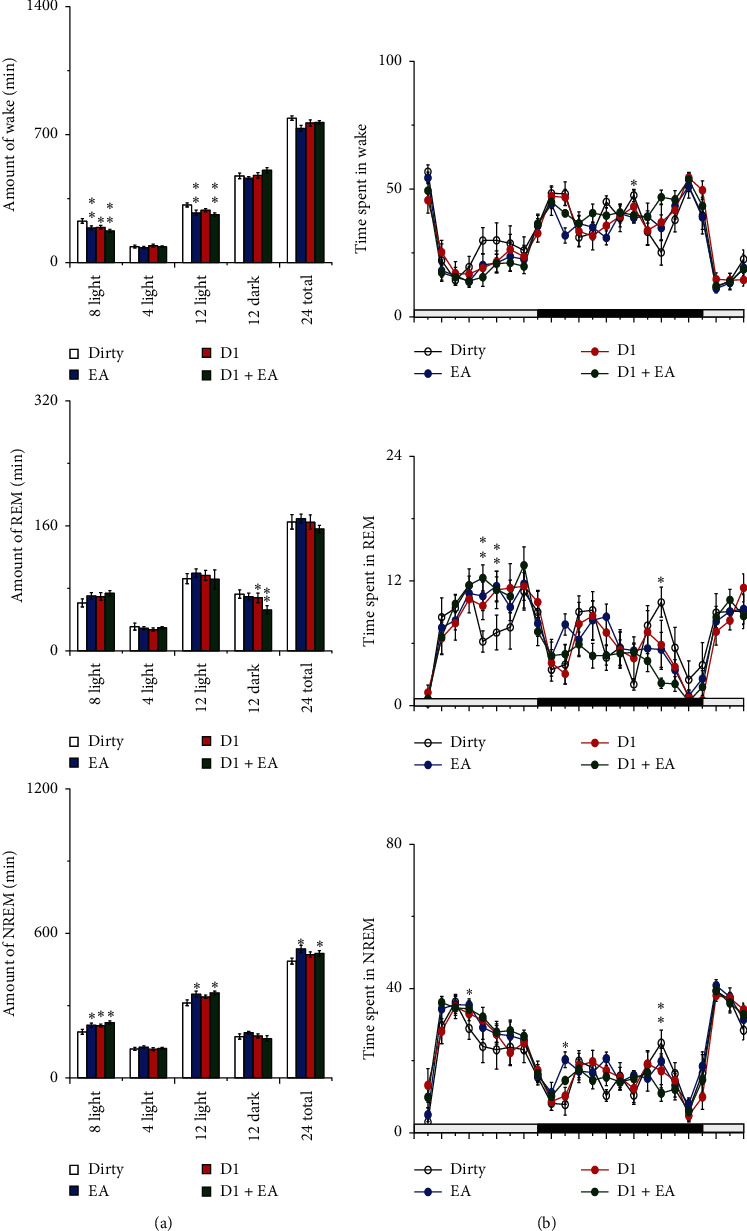
Sleep-wake profiles of the four groups after intervention. (a) Total time spent in wakefulness, NREM, and REM sleep during the 8 h light, 4 h light, 12 h light, 12 h dark, and 24 h total periods, respectively. Open bars, blue filled bars, red filled bars, and green filled bars show the profiles for the dirty group, EA group, D1 group, and D1 + EA group, respectively. (b) Time course changes in wakefulness, NREM, and REM sleep. Each circle represents the hourly mean amount of each stage. Open circles, blue filled circles, red filled circles, and green filled circles represent the dirty group, EA group, D1 group, and D1 + EA group, respectively. The horizontal open and filled bars on the axes indicate the light and dark periods, respectively. Values are the mean ± SEM (*n* = 7∼9). Compared with the dirty group, ^*∗*^*P* < 0.05, ^*∗∗*^*P* < 0.01. Comparisons were performed using analysis of variance of factorial design (2 × 2 factorial design).

The main effect of EA and the D1R antagonist on the amount of wakefulness and NREM sleep was statistically significant during the 8 h light period (*P* < 0.05); compared with those of the dirty group, the amounts of wakefulness decreased and NREM sleep increased during the 8 h light period in the EA, D1, and D1 + EA groups (*P* < 0.05). The main effect of EA on the amount of wakefulness and NREM sleep was statistically significant during the 12 h light period (*P* < 0.05); compared with those of the dirty group, the amounts of wakefulness decreased and NREM sleep increased during the 12 h light period in the EA and D1 + EA groups (*P* < 0.05). The main effect of the D1R antagonist on the amount of REM sleep was statistically significant during the 12 h dark period (*P* < 0.05); compared with the dirty group, the amounts of REM sleep decreased during the 12 h dark period in the D1 and D1 + EA groups (*P* < 0.05). The main effect of EA on the amount of NREM sleep was statistically significant in 24 h (*P* < 0.05); compared with the dirty group, the amount of NREM sleep increased in 24 h in the EA and D1 + EA groups, and the differences were statistically significant (*P* < 0.05). See Figure 7 and Table 1 for details.

*(3) The Amounts of Sleep-Wake among the Dirty, EA, D2, and D2 + EA groups*. There was no interaction between EA and the D2R antagonist in the amounts of sleep-wake (*P* > 0.05), which indicated that the combined application of EA and the D2R antagonist did not have a better effect on the amounts of sleep-wake than a single application. See Table 2 for details.

**Figure 8:**
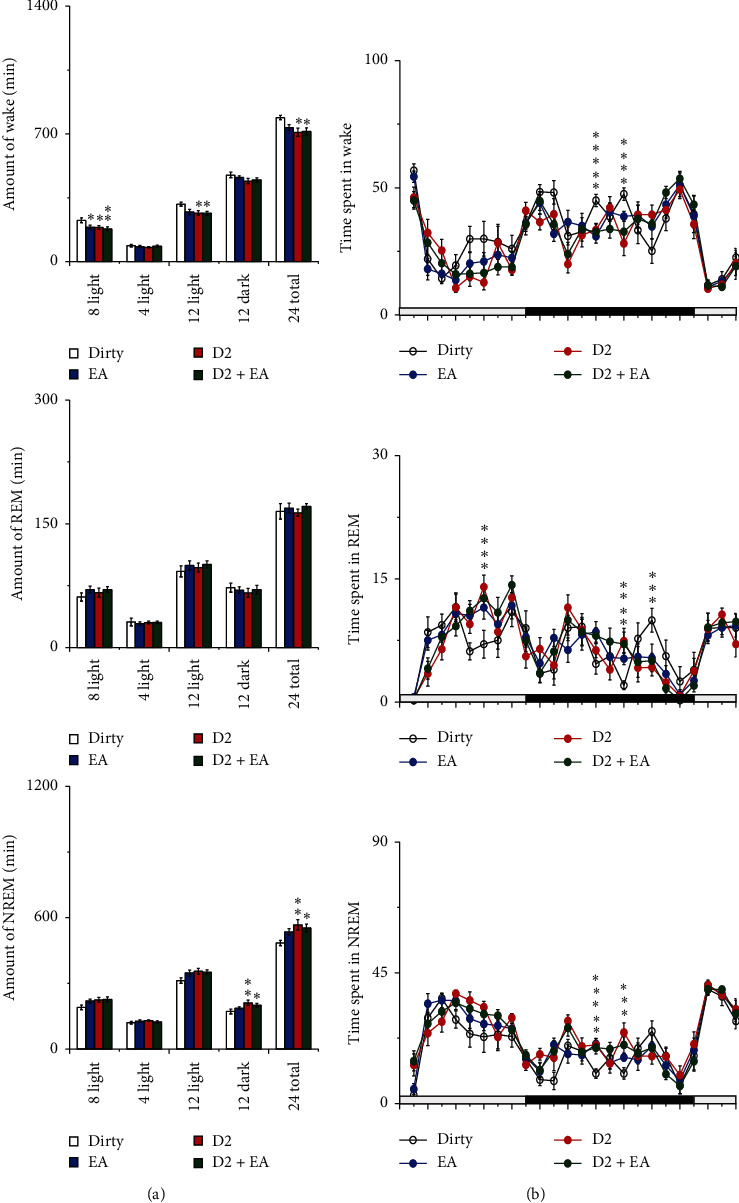
Sleep-wake profiles of the four groups after intervention. (a) Total time spent in wakefulness, NREM, and REM sleep during the 8 h light, 4 h light, 12 h light, 12 h dark, and 24 h total periods. Open bars, blue filled bars, red filled bars, and green filled bars show the profiles for the dirty group, EA group, D2 group, and D2 + EA group, respectively. (b) Time course changes in wakefulness, NREM, and REM sleep. Each circle represents the hourly mean amount of each stage. Open circles, blue filled circles, red filled circles, and green filled circles represent the dirty group, EA group, D2 group, and D2 + EA group, respectively. The horizontal open and filled bars on the axes indicate the light and dark periods, respectively. Values are the mean ± SEM (*n* = 7–9). Compared with the dirty group, ^*∗*^*P* < 0.05, ^*∗∗*^*P* < 0.01. Comparisons were performed using analysis of variance of factorial design (2 × 2 factorial design).

The main effect of EA and the D2R antagonist on the amount of wakefulness was statistically significant during the 8 h light period (*P* < 0.05); compared with those of the dirty group, the amounts of wakefulness decreased during the 8 h light period in the EA, D2, and D2 + EA groups (*P* < 0.05). The main effect of the D2R antagonist on the amount of wakefulness was statistically significant during the 12 h light period (*P* < 0.05); compared with that of the dirty group, the amount of wakefulness decreased during the 12 h light period in the D2 and D2 + EA groups (*P* < 0.05). The main effect of the D2R antagonist on the amount of NREM sleep was statistically significant during the 12 h dark period (*P* < 0.05); compared with that of the dirty group, the amount of NREM sleep increased during the 12 h dark period in the D2 and D2 + EA groups (*P* < 0.05). The main effect of the D2R antagonist on the amount of wakefulness and NREM sleep was statistically significant in the 24 h (*P* < 0.05); compared with that of the dirty group, the amounts of wakefulness decreased and NREM sleep increased in 24 hours in the D2 and D2 + EA groups, and the differences were statistically significant (*P* < 0.05). See Figure 8 and Table 2 for details.

#### 3.2.2. Effect of EA on the Numbers of Sleep Bouts and Episodes

**Figure 9:**
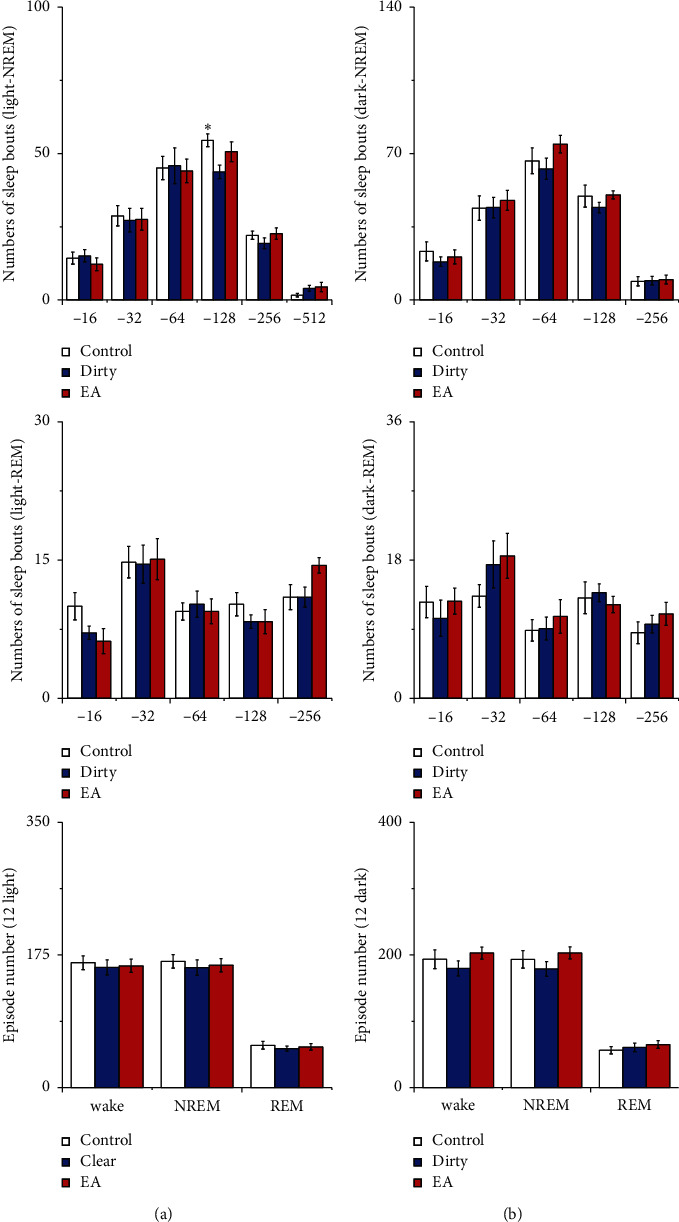
(a) Numbers of sleep bouts and (b) episode numbers. Open bars, blue filled bars, and red filled bars show the profiles for the control group, dirty group, and EA group, respectively. Values are the mean ± SEM (*n* = 7∼9). Compared with the dirty group, ^*∗*^*P* < 0.05, ^*∗∗*^*P* < 0.01. Comparisons were performed using one-way ANOVA followed by Fisher's PLSD for each planned comparison.

Compared with those of the control group, the numbers of long NREM sleep bouts during the light period decreased in the dirty group (*P* < 0.05). There was no statistically significant difference between the EA group and the dirty group in the numbers of sleep bouts and episodes (*P* > 0.05). See Figure 9 for details.

**Figure 10:**
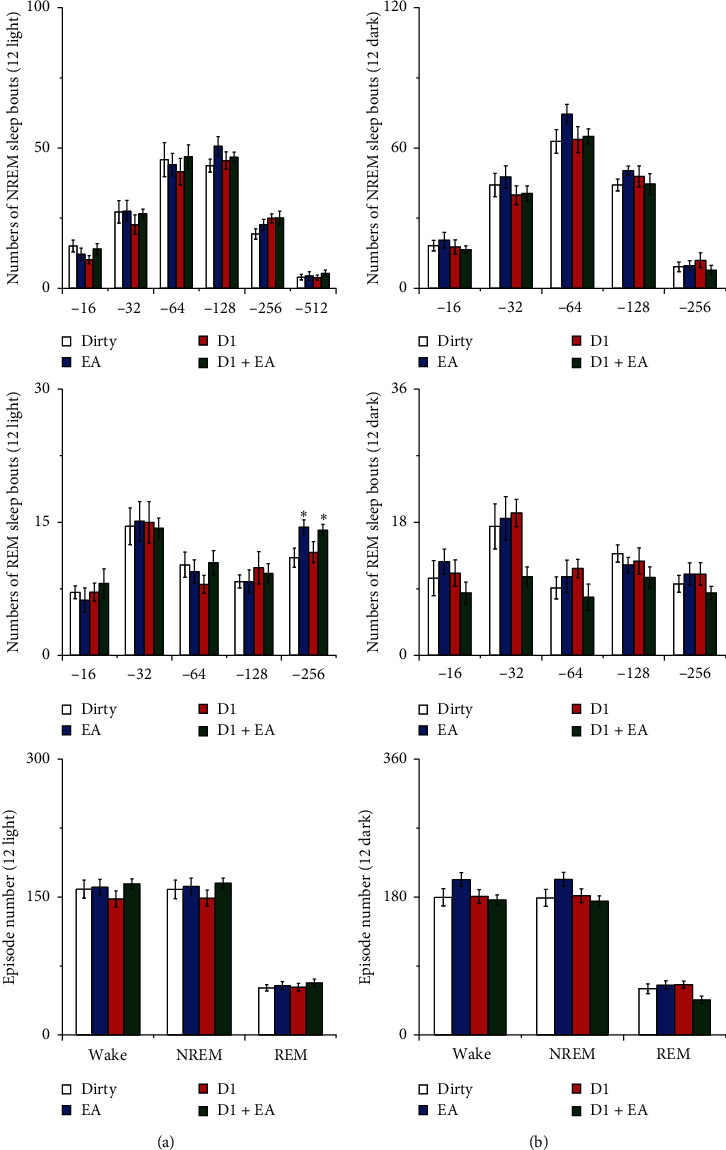
(a) Numbers of sleep bouts and (b) episode numbers. Open bars, blue filled bars, red filled bars, and green filled bars show the profiles for the dirty group, EA group, D1 group, and D1 + EA group, respectively. Values are the mean ± SEM (*n* = 7–9). Compared with the dirty group, ^*∗*^*P* < 0.05, ^*∗∗*^*P* < 0.01. Comparisons were performed using analysis of variance of factorial design (2 × 2 factorial design).

There was no interaction between EA and the D1R antagonist in the numbers of sleep bouts and episodes (*P* > 0.05), which indicated that the combined application of EA and the D1R antagonist did not have a better effect on the numbers of sleep bouts and episodes than a single application. The main effect of EA on the numbers of long REM sleep bouts (256 s) was statistically significant during the 12 h light period (*P* < 0.05); compared with that of the dirty group, the amount of wakefulness decreased during the 12 h light period in the D2 and D2 + EA groups (*P* < 0.05). See Figure 10 for details.

**Figure 11:**
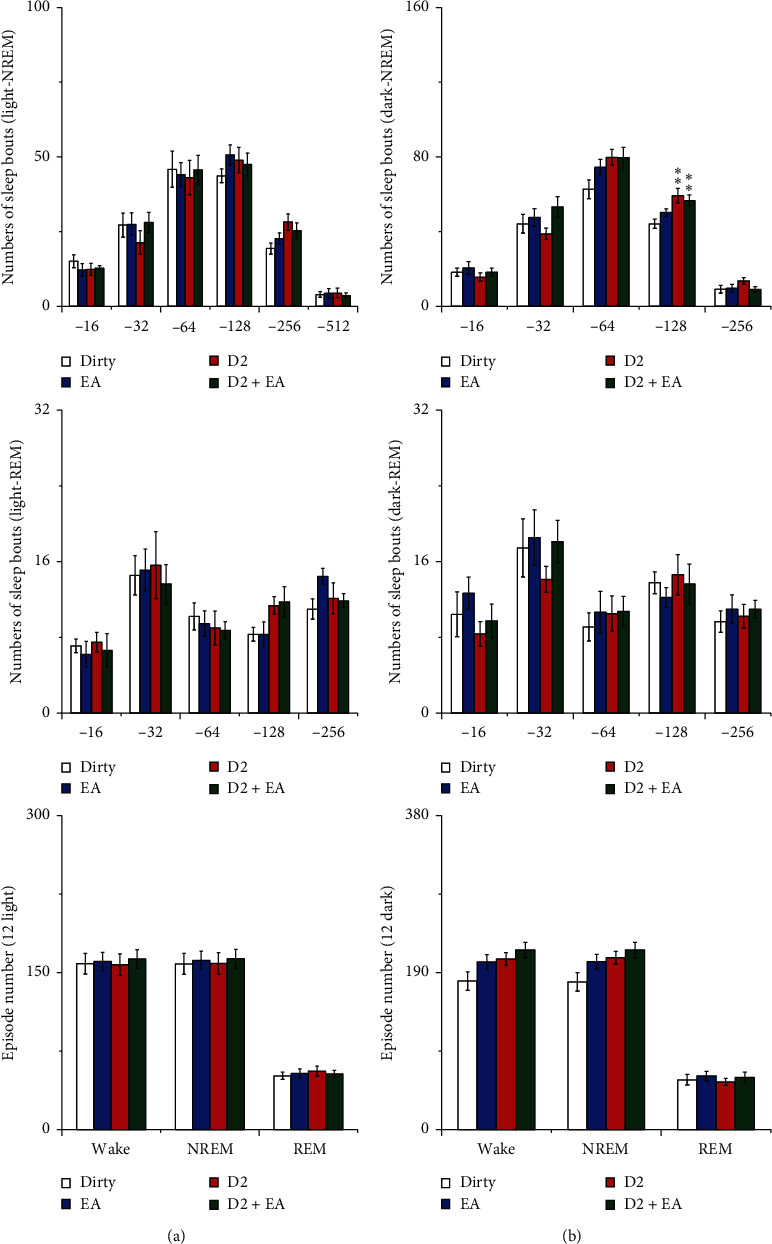
(a) Numbers of sleep bouts and (b) episode numbers. Open bars, blue filled bars, red filled bars, and green filled bars show the profiles for the dirty group, EA group, D2 group, and D2 + EA group, respectively. Values are the mean ± SEM (*n* = 7–9). Compared with the dirty group, ^*∗*^*P* < 0.05, ^*∗∗*^*P* < 0.01. Comparisons were performed using analysis of variance of factorial design (2 × 2 factorial design).

There was no interaction between EA and the D2R antagonist in the numbers of sleep bouts and episodes (*P* > 0.05), which indicated that the combined application of EA and the D2R antagonist did not have a better effect on the numbers of sleep bouts and episodes than a single application. The main effect of the D2R antagonist on the numbers of long NREM sleep bouts (256 s) were statistically significant during the 12 h dark period (*P* < 0.05); compared with that of the dirty group, the numbers of long NREM bouts increased during the 12 h dark period in D2 and D2 + EA groups (*P* < 0.05). See Figure 11 for details.

#### 3.2.3. Effect of EA on the Mean Durations of Wakefulness, NREM, and REM

**Figure 12:**
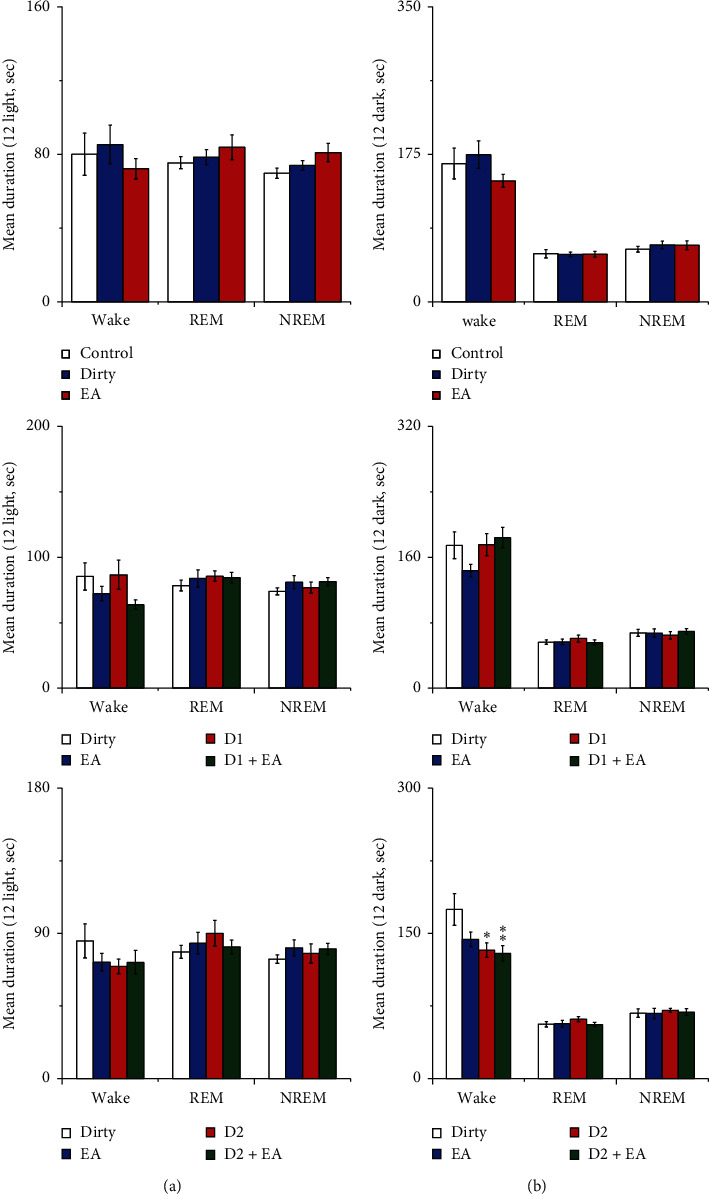
(a) Mean duration during the 12 h light period; (b) mean duration during the 12 h dark period. Open bars, blue filled bars, and red filled bars show the profiles for the control group, dirty group, and EA group, respectively. Open bars, blue filled bars, red filled bars, and green filled bars show the profiles for the dirty group, EA group, D1 group, and D1 + EA group, respectively. Open bars, blue filled bars, red filled bars, and green filled bars show the profiles for the dirty group, EA group, D2 group, and D2 + EA group, respectively. Values are the mean ± SEM (*n* = 7∼9). Compared with the dirty group, ^*∗*^*P* < 0.05, ^*∗∗*^*P* < 0.01. Comparisons were performed using one-way ANOVA followed by Fisher's PLSD for each planned comparison and analysis of variance of factorial design (2 × 2 factorial design).

There was no interaction between EA and the D2R antagonist in the mean durations of wakefulness, NREM, and REM (*P* > 0.05), which indicated that the combined application of EA and the D2R antagonist did not have a better effect on mean durations of wakefulness, NREM, and REM than a single application. The main effect of the D2R antagonist on the mean duration of wakefulness was statistically significant during the 12 h dark period (*P* < 0.05); compared with that of the dirty group, the mean duration of wakefulness during the dark period decreased in the D2 and EA + D2 groups (*P* < 0.05). See Figure 12 for details.

#### 3.2.4. Effect of EA on the Stage Transition Numbers from Wakefulness, NREM, and REM

**Figure 13:**
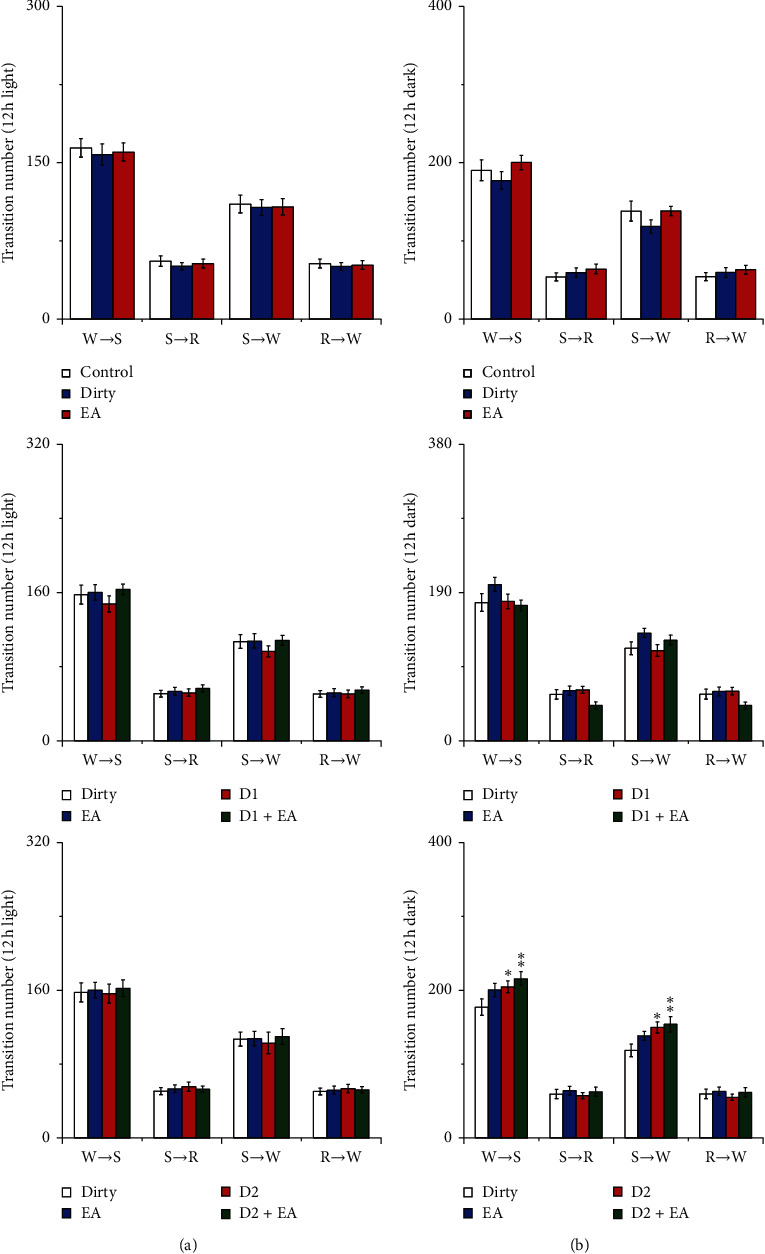
(a) Transition numbers during the 12 h light period; (b) transition numbers during the 12 h dark period. Open bars, blue filled bars, and red filled bars show the profiles for the control group, dirty group, and EA group, respectively. Open bars, blue filled bars, red filled bars, and green filled bars show the profiles for the dirty group, EA group, D1 group, and D1 + EA group, respectively. Open bars, blue filled bars, red filled bars, and green filled bars show the profiles for the dirty group, EA group, D2 group, and D2 + EA group, respectively. Values are the mean ± SEM (*n* = 7–9). Compared with the dirty group, ^*∗*^*P* < 0.05, ^*∗∗*^*P* < 0.01. Comparisons were performed using one-way ANOVA followed by Fisher's PLSD for each planned comparison and analysis of variance of factorial design (2 × 2 factorial design).

There was no interaction between EA and the D2R antagonist in the stage transition numbers from wakefulness, NREM, and REM (*P* > 0.05), which indicated that the combined application of EA and the D2R antagonist did not have a better effect on the stage transition numbers from wakefulness, NREM, and REM than that of a single application. The main effect of the D2R antagonist on the transition numbers from wakefulness to NREM sleep and from NREM sleep to wakefulness was statistically significant during the 12 h dark period (*P* < 0.05); compared with those of the dirty group, the transition numbers from wakefulness to NREM sleep and from NREM sleep to wakefulness during the 12 h dark period increased in the D2 and EA + D2 groups (*P* < 0.05). See Figure 13 for details.

### 3.3. Effect of EA on the Levels of Serum and Hypothalamus DA, Corticotropin-Releasing Hormone (CRH), Adrenocorticotropic Hormone (ACTH), and Cortisol (CORT)

There was no statistically significant difference in the levels of serum DA, CRH, ACTH, and CORT among the seven groups 24 hours before intervention (*P* > 0.05).

**Figure 14:**
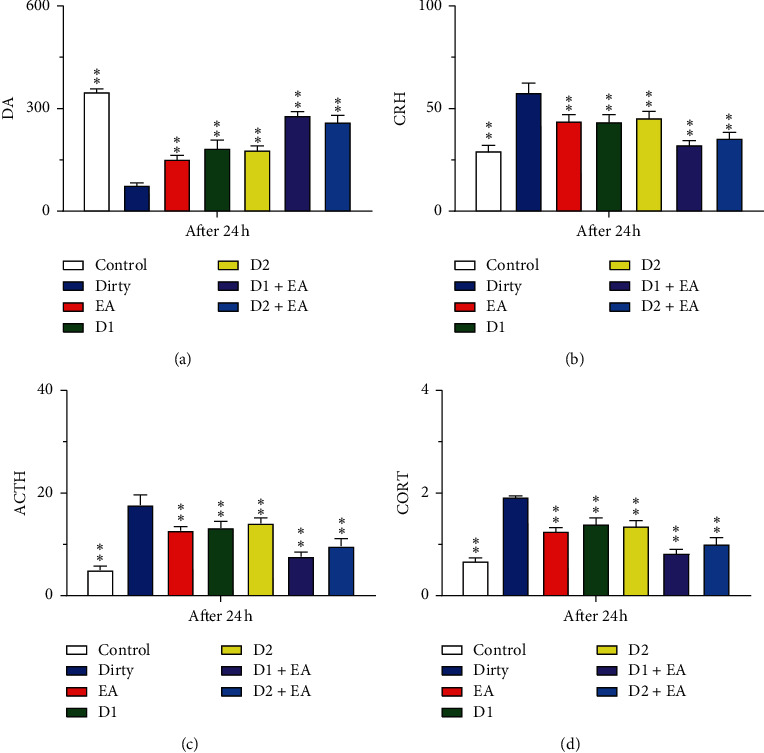
(a) The content of DA in the hypothalamus. (b) The content of CRH in the hypothalamus. (c) The content of ACTH in the hypothalamus. (d) The content of CORT in the hypothalamus. Open bars, blue filled bars, red filled bars, green filled bars, yellow filled bars, purple filled bars, and sky blue filled bars show the profiles for the dirty group, EA group, D1 group, D2 group, D1 + EA group, and D2 + EA group, respectively. Values are the mean ± SEM (*n* = 7–9). Compared with the dirty group, ^*∗*^*P* < 0.05, ^*∗∗*^*P* < 0.01. Comparisons were performed using one-way ANOVA followed by Fisher's PLSD for each planned comparison.

The levels of DA, CRH, ACTH, and CORT in the hypothalamus of the dirty group were lower than those in the control group (*P* < 0.05). After EA intervention, the levels of DA, CRH, ACTH, and CORT in the hypothalamus increased compared with those in the dirty group (*P* < 0.05). The main effect of EA and the D1R (or D2R) antagonist on the levels of DA, CRH, ACTH, and CORT in the hypothalamus was statistically significant (*P* < 0.05); compared with those of the dirty group, the levels of DA, CRH, ACTH, and CORT in the hypothalamus increased after D1R (or D2R) antagonist and EA intervention (*P* < 0.05). There was no interaction between EA and the D1R (or D2R) antagonist in the levels of DA, CRH, ACTH, and CORT in the hypothalamus (*P* > 0.05), which indicated that the combined application of EA and the D1R (or D2R) antagonist did not have a better effect on the levels of DA, CRH, ACTH, and CORT in the hypothalamus than a single application. See Figure 14 and Tables 3 and 4 for details.

### 3.4. Effect of EA on the Expression of D1R and D2R in the HPA Axis

**Figure 15:**
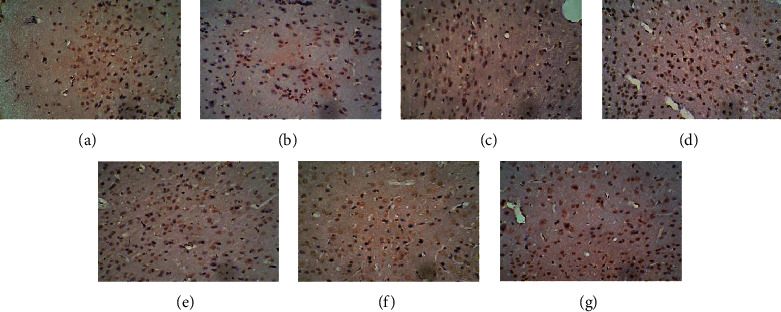
Expression of D1R in the hypothalamus among the seven groups. Cells stained for hematoxylin showed brown cell membranes with blue or brown nuclei and were D1R-positive cells. Scale bar: 200 *μ*m, under the light microscope. (a) Control. (b) Dirty. (c) EA. (d) D1. (e) D1 + EA. (f) D2. (g) D2 + EA.

**Figure 16:**
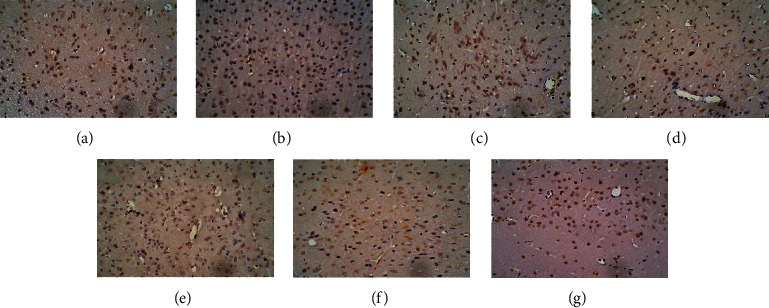
Expression of D2R in the hypothalamus among the seven groups. Cells stained for hematoxylin showed brown cell membranes with blue or brown nuclei and were D2R-positive cells. Scale bar: 200 *μ*m, under the light microscope. (a) Control. (b) Dirty. (c) EA. (d) D1. (e) D1 + EA. (f) D2. (g) D2 + EA.

**Figure 17:**
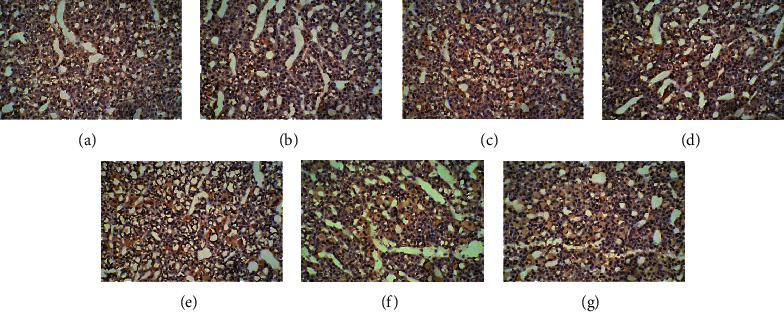
Expression of D1R in the pituitary among the seven groups. Cells stained for hematoxylin showed brown cell membranes with blue or brown nuclei and were D1R-positive cells. Scale bar: 200 *μ*m, under the light microscope. (a) Control. (b) Dirty. (c) EA. (d) D1. (e) D1 + EA. (f) D2. (g) D2 + EA.

**Figure 18:**
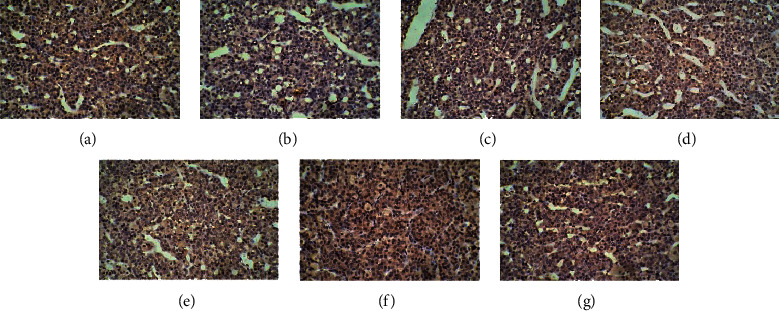
Expression of D2R in the pituitary among the seven groups. Cells stained for hematoxylin showed brown cell membranes with blue or brown nuclei and were D2R-positive cells. Scale bar: 200 *μ*m, under the light microscope. (a) Control. (b) Dirty. (c) EA. (d) D1. (e) D1 + EA. (f) D2. (g) D2 + EA.

**Figure 19:**
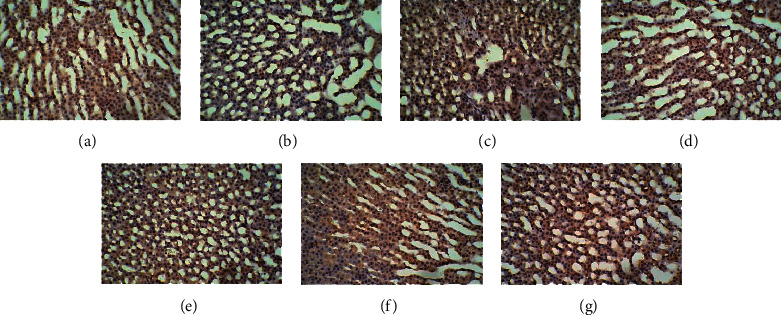
Expression of D1R in the adrenal gland among the seven groups. Cells stained for hematoxylin showed brown cell membranes with blue or brown nuclei and were D1R-positive cells. Scale bar: 200 *μ*m, under the light microscope. (a) Control. (b) Dirty. (c) EA. (d) D1. (e) D1 + EA. (f) D2. (g) D2 + EA.

**Figure 20:**
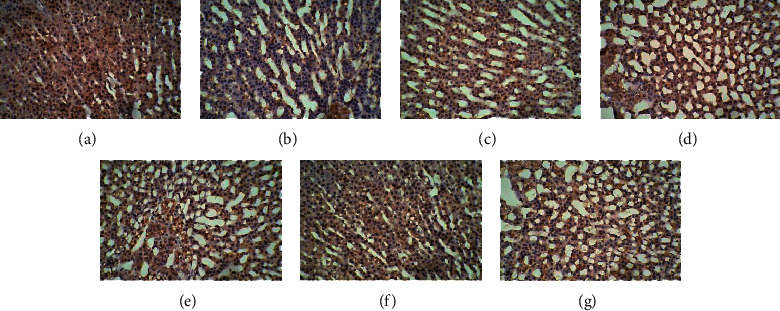
Expression of D2R in the adrenal gland among the seven groups. Cells stained for hematoxylin showed brown cell membranes with blue or brown nuclei and were D2R-positive cells. Scale bar: 200 *μ*m, under the light microscope. (a) Control. (b) Dirty. (c) EA. (d) D1. (e) D1 + EA. (f) D2. (g) D2 + EA.

In the control group, many cells with brown cell membranes were found in the hypothalamus, pituitary, and adrenal gland, while the number of cells with brown cell membranes decreased in the dirty group. The number of cells with brown cell membranes in the hypothalamus, pituitary, and adrenal gland was increased after EA and dopamine receptor antagonist intervention (Figures 15–20).

### 3.5. Effect of EA on the mRNA Levels of D1R and D2R in the HPA Axis

**Figure 21:**
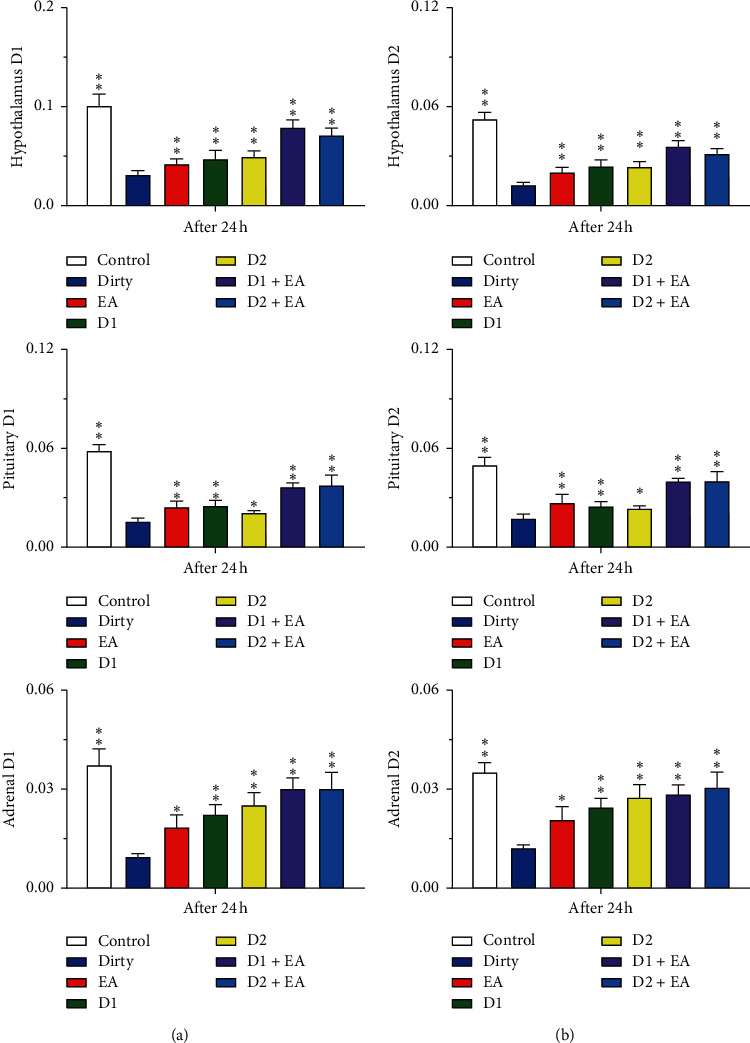
(a) The level of D1R mRNA in the hypothalamus, pituitary, and adrenal gland. (b) The level of D2R mRNA in the hypothalamus, pituitary, and adrenal gland. Open bars, blue filled bars, red filled bars, green filled bars, yellow filled bars, purple filled bars, and sky blue filled bars show the profiles for the dirty group, EA group, D1 group, D2 group, D1 + EA group, and D2 + EA group, respectively. Values are the mean ± SEM (*n* = 7–9). Compared with the dirty group, ^*∗*^*P* < 0.05, ^*∗∗*^*P* < 0.01. Comparisons were performed using one-way ANOVA followed by Fisher's PLSD for each planned comparison.

The levels of D1R and D2R mRNA in the hypothalamus, pituitary, and adrenal gland in the dirty group were lower than those in the control group (*P* < 0.05). After electroacupuncture intervention, the mRNA levels of D1R and D2R in the hypothalamus, pituitary, and adrenal gland increased compared with those of the dirty group (*P* < 0.05). The main effect of EA and D1R (or D2R) antagonist on the levels of D1R and D2R mRNA in the hypothalamus was statistically significant (*P* < 0.05); compared with those of the dirty group, the levels of D1R and D2R mRNA in the hypothalamus increased after D1R (or D2R) antagonist and EA intervention (*P* < 0.05). There was no interaction between EA and the D1R (or D2R) antagonist in the levels of D1R and D2R mRNA in the hypothalamus (*P* > 0.05), which indicated that the combined application of EA and the D1R (or D2R) antagonist did not have a better effect on the levels of D1R and D2R mRNA in the hypothalamus than a single application. See Figure 21 and Tables 5 and 6 for details.

## 4. Discussion

### 4.1. Sleep and Wake

The cage change model of insomnia (rodent model of acute insomnia) is often used to simulate stress-induced insomnia in humans [38, 39]. During the peak period of sleep in rats, at 10:00 AM, rats were moved into a dirty cage occupied by another male rat, which would activate their defensive nature, promote their fight or flight response, and then affect their sleep-wake pathway, resulting in an increase in the frequency of awakening-related waves in EEG, similar to sleep perturbations induced by stress.

The rats after cage change exhibited a significant increase in wakefulness concomitant with a decrease in NREM sleep during the light period and an increase in REM sleep during the night period. Moreover, the sleep structure changed, showing a decrease in the numbers of long-term NREM bouts during the light period. After EA intervention, wakefulness decreased, NREM increased during the light period, and the sleep structure did not change significantly in the cage change model. After D1R antagonist intervention, the rats that underwent cage change exhibited a significant decrease in wakefulness, an increase in NREM sleep after cage change during the light period, and a decrease in REM sleep during the night period.

After D1R antagonist intervention, the numbers of long-term REM sleep events increased in the D1 group, which was significantly different from that in the dirty group (*P* < 0.05); the numbers of long-term NREM sleep events increased in the D1 group, but there was no statistically significant difference compared with the dirty group (*P* > 0.05). Sleep-wake changes in the D1 group during the light period were consistent with the existing results. Previous studies only focused on the amount of sleep-wake over several hours during the light period after the cage change [40, 41]. However, this study found that the cage change group after D1R antagonist intervention showed a decrease in REM sleep during the night period, suggesting that D1R antagonists can also affect REM sleep during the night period in the cage change group.

This study found that wakefulness decreased during the light period, wakefulness decreased, NREM increased during 24 h total, and NREM sleep increased during the 12 h dark period in the cage change group after D2R antagonist intervention. After D2R antagonist intervention, the number of long-term NREM sleep events during the night period increased in the D2 group, which was significantly different from that in the dirty group (*P* < 0.05); the number of long-term NREM sleep events increased in the D2 group, but there was no statistically significant difference compared with the dirty group (*P* > 0.05). The mean duration of wakefulness during the night period in the D2 group decreased, which was significantly different from that in the dirty group (*P* < 0.05). The mean duration of NREM sleep during the light period in the D2 group increased, but there was no significant difference compared with the dirty group (*P* > 0.05). The S-W transition numbers increased in the D2 group during the night period, which was significantly different from that in the dirty group (*P* < 0.05). The sleep-wake change in the cage change rats after D2R antagonist intervention is not consistent with the existing research results [32]. The reason may be that the author chose rats, while the current research used mice.

The factorial analysis showed that EA and D1R antagonists could improve sleep-wake cycles in the cage change group. The combination of EA and the D1R antagonist did not improve sleep in rats better than a single treatment. Factorial analysis of EA and D2R antagonist showed that EA could reduce the amount of sleep-wake during the 8 h light period but had no effect on other sleep quantities, while D2R antagonist had effects on the sleep quantities during the 12 h light period, 12 h dark period, and 24 h total, especially NREM sleep during the 12 h dark period. However, EA had no influence on wakefulness and sleep during the 12 h dark period in the cage change group, which suggests that EA can improve sleep by regulating the amount of sleep during the light period after cage change without affecting sleep during the night period in the cage change group.

### 4.2. DA, DA Receptors, and Sleep-Wake Cycles

Dopamine is associated with sleep-wake cycles and increases during wakefulness [42, 43]. Dopamine- (DA-) containing neurons involved in the regulation of sleep and waking (W) arise in the ventral tegmental area (VTA) and the substantia nigra pars compacta (SNc) [44]. DA receptors include two families of dopamine receptors, D1 and D2-like receptors [45]. The D1 receptor (D1R) and D2 receptor (D2R) both mediate sleep and wakefulness by D1R and D2R agonists and antagonists [46]. The sleep in the cage change group may be improved by dopamine receptor antagonists [47].

In this study, we found that the level of dopamine in the hypothalamus decreased 24 hours after cage change compared with that of the control group. The decrease in the level of dopamine in the dirty group during the 24 hours after cage change may be due to the increase in sleep pressure compared with that of the no cage change group without sleep pressure. After EA and/or DA receptor antagonist intervention, the level of dopamine in the cage change group increased. The level of dopamine receptor mRNA in the HPA axis decreased 24 hours after the cage change, which may be due to the decreased level of dopamine. After EA and/or dopamine receptor antagonist intervention, dopamine receptor antagonists bound to dopamine receptors; as a result, the level of free dopamine increased, which stimulated dopamine receptor mRNA secretion in the HPA axis, which is consistent with the current research results [47]. In addition, the effect of EA combined with dopamine receptor antagonists is not superior to that of single use.

Dopamine receptor antagonists had no effect on DA levels in the peripheral blood of the cage change group. Immunohistochemistry results showed that the expression of dopamine receptors in the HPA axis decreased after the cage change but increased after the intervention with dopamine receptor antagonists, which was consistent with the mRNA levels.

### 4.3. The HPA Axis and Sleep-Wake Cycles

Stress may be an important factor causing insomnia [17, 48]. Acupuncture has a significant effect on stress reactions [49]. Acupuncture is effective in reducing posttraumatic stress syndrome and improving depression, pain, psychological, and physiological dysfunction [50]. Acupuncture or EA also inhibited the activity of the HPA axis and reduce the plasma CORT content in rats with chronic stress anxiety [34, 51]. EA significantly reduced the expression of CRH and CRHR1 mRNA in the paraventricular nucleus of the hypothalamus in a rat model of stress-induced anxiety [52]. EA reduced brain ACh, CRH, serum ACTH, and CORT in rats with insomnia under psychological stress and then regulated the activity of the HPA [53]. Here, we found that EA and/or dopamine receptor antagonists could reduce the levels of CRH, ACTH, and CORT in the hypothalamus 24 hours after the cage change, indicating that EA and/or dopamine receptor antagonists could improve the function of the HPA axis. In addition, the effect of EA combined with dopamine receptor antagonists is not superior to that of single use.

In conclusion, we found here that EA could increase DA in the hypothalamus, increase D1R and D2R in the HPA axis, and decrease CRH, ACTH, and CORT in the hypothalamus in rats after cage change, suggesting that EA may regulate hypothalamus DA and DA receptors in the HPA axis and alleviate changes in neurotransmitters induced by stress to improve sleep-wake cycles in rats with acute insomnia. Furthermore, EA intervention had a similar effect to DA receptor antagonist on the hypothalamic neurotransmitter and the DA receptors in the HPA axis in rats with acute insomnia, and the combined application of EA and DA receptor antagonist is not better than single treatment. EA could improve sleep-wake cycles during the light period in the cage change group without change in the sleep structure in the dark period, and the effect of EA was not better than that of the dopamine receptor antagonist alone, suggesting that EA could be used in the treatment of insomnia without using drugs.

## Figures and Tables

**Table 1:** Factorial analysis of the amounts of sleep-wake after intervention with EA and a D1R antagonist.

Factor	*P* (8 h light period)			*P* (12 h light period)			*P* (12 h dark period)			*P* (24 hours)		
	Wake	REM	NREM	Wake	REM	NREM	Wake	REM	NREM	Wake	REM	NREM
EA (A)	0.009	0.139	0.014	0.007	0.171	0.018	0.562	0.066	0.837	0.079	0.794	0.039
D1 (B)	0.029	0.202	0.045	0.126	0.379	0.207	0.102	0.034	0.265	0.819	0.377	0.826
EA-D1 (A-B)	0.460	0.583	0.422	0.510	0.893	0.422	0.136	0.200	0.170	0.068	0.410	0.106

**Table 2:** Factorial analysis of the amounts of sleep-wake after intervention with EA and a D2R antagonist.

Factor	*P* (8 h light period)			*P* (12 h light period)			*P* (12 h dark period)			*P* (24 hours)		
	Wake	REM	NREM	Wake	REM	NREM	Wake	REM	NREM	Wake	REM	NREM
EA (A)	0.049	0.197	0.154	0.097	0.321	0.221	0.809	0.931	0.789	0.186	0.318	0.320
D2 (B)	0.037	0.504	0.060	0.042	0.581	0.077	0.056	0.555	0.006	0.010	0.992	0.010
EA-D2 (A-B)	0.196	0.511	0.237	0.117	0.776	0.129	0.420	0.491	0.151	0.109	0.745	0.077

**Table 3:** Factorial analysis of the levels of DA, CRH, ACTH, and CORT in the hypothalamus after treatment with EA and D1R antagonists.

Factor	*P*			
	DA	CRH	ACTH	CORT
EA (A)	0.000	0.001	0.000	0.000
D1 (B)	0.000	0.001	0.001	0.000
EA-D1 (A-B)	0.499	0.718	0.797	0.538

**Table 4:** Factorial analysis of the levels of DA, CRH, ACTH, and CORT in the hypothalamus by after treatment with EA and D2R antagonists.

Factor	*P*			
	DA	CRH	ACTH	CORT
EA (A)	0.000	0.002	0.002	0.000
D2 (B)	0.000	0.006	0.020	0.000
EA-D2 (A-B)	0.829	0.589	0.866	0.084

**Table 5:** Factorial analysis of D1R and D2R mRNA in the HPA axis after treatment with EA and D1R antagonists.

Factor	*P* (D1R)			*P* (D2R)		
	Hypothalamus	Pituitary	Adrenal	Hypothalamus	Pituitary	Adrenal
EA (A)	0.004	0.001	0.003	0.003	0.000	0.033
D1 (B)	0.001	0.001	0.000	0.000	0.003	0.001
EA-D1 (A-B)	0.137	0.643	0.858	0.477	0.355	0.413

**Table 6:** Factorial analysis of D1R and D2R mRNA in the HPA axis after treatment with EA and D2R antagonists.

Factor	*P* (D1R)			*P* (D2R)		
	Hypothalamus	Pituitary	Adrenal	Hypothalamus	Pituitary	Adrenal
EA (A)	0.008	0.001	0.028	0.007	0.004	0.074
D2 (B)	0.000	0.017	0.009	0.000	0.027	0.000
EA-D2 (A-B)	0.359	0.290	0.258	0.968	0.388	0.263

## Data Availability

The data supporting the results of this study are publicly available.

## References

[1] Dopheide J. A. (2020). Insomnia overview: epidemiology, pathophysiology, diagnosis and monitoring, and nonpharmacologic therapy. *The American Journal of Managed Care*.

[2] Chung K.-F., Yeung W.-F., Ho F. Y.-Y., Yung K.-P., Yu Y.-M., Kwok C.-W. (2015). Cross-cultural and comparative epidemiology of insomnia: the diagnostic and statistical manual (DSM), international classification of diseases (ICD) and international classification of sleep disorders (ICSD). *Sleep Medicine*.

[3] Roth T., Coulouvrat C., Hajak G. (2011). Prevalence and perceived health associated with insomnia based on DSM-IV-TR; international statistical classification of diseases and related health problems, tenth revision; and research diagnostic criteria/international classification of sleep disorders, second edition criteria: results from the America insomnia survey. *Biological Psychiatry*.

[4] Schutterodin S., Broch L., Buysse D., Dorsey C., Sateia M. (2008). Clinical guideline for the evaluation and management of chronic insomnia in adults. *Journal of Clinical Sleep Medicine*.

[5] Moloney M. E., Ciciurkaite G., Brown R. L. (2019). The medicalization of sleeplessness: results of U.S. office visit outcomes, 2008-2015. *SSM-Population Health*.

[6] Wickwire E. M., Tom S. E., Scharf S. M., Vadlamani A., Bulatao I. G., Albrecht J. S. (2019). Untreated insomnia increases all-cause health care utilization and costs among medicare beneficiaries. *Sleep*.

[7] Morin C. M., Drake C. L., Harvey A. G. (2015). Insomnia disorder. *Nature Reviews Disease Primers*.

[8] Nishitani N., Kawasaki Y., Sakakibara H. (2018). Insomnia and depression: risk factors for development of depression in male Japanese workers during 2011–2013. *International Journal of Public Health*.

[9] Bragantini D., Sivertsen B., Gehrman P., Lydersen S., Güzey I. C. (2019). Differences in anxiety levels among symptoms of insomnia. The hunt study. *Sleep Health*.

[10] Sofi F., Cesari F., Casini A., Macchi C., Abbate R., Gensini G. F. (2014). Insomnia and risk of cardiovascular disease: a meta-analysis. *European Journal of Preventive Cardiology*.

[11] Bertisch S. M., Pollock B. D., Mittleman M. A. (2018). Insomnia with objective short sleep duration and risk of incident cardiovascular disease and all-cause mortality: sleep heart health study. *Sleep*.

[12] Bernert R. A., Kim J. S., Iwata N. G., Perlis M. L. (2015). Sleep disturbances as an evidence-based suicide risk factor. *Current Psychiatry Reports*.

[13] Parthasarathy S., Vasquez M. M., Halonen M. (2015). Persistent insomnia is associated with mortality risk. *The American Journal of Medicine*.

[14] Garbarino S., Magnavita N., Guglielmi O. (2017). Insomnia is associated with road accidents. further evidence from a study on truck drivers. *PLoS One*.

[15] Palagini L., Biber K., Riemann D. (2014). The genetics of insomnia - evidence for epigenetic mechanisms?. *Sleep Medicine Reviews*.

[16] Riemann D., Spiegelhalder K., Feige B. (2010). The hyperarousal model of insomnia: a review of the concept and its evidence. *Sleep Medicine Reviews*.

[17] Almojali A. I., Almalki S. A., Alothman A. S., Masuadi E. M., Alaqeel M. K. (2017). The prevalence and association of stress with sleep quality among medical students. *Journal of Epidemiology and Global Health*.

[18] Wang T., Wang H.-L., Liu R. (2019). Early-life stress alters sleep structure and the excitatory-inhibitory balance in the nucleus accumbens in aged mice. *Chinese Medical Journal*.

[19] Pillai V., Roth T., Mullins H. M., Drake C. L. (2014). Moderators and mediators of the relationship between stress and insomnia: stressor chronicity, cognitive intrusion, and coping. *Sleep*.

[20] Buckley T. M., Schatzberg A. F. (2005). On the interactions of the hypothalamic-pituitary-adrenal (HPA) axis and sleep: normal HPA axis activity and circadian rhythm, exemplary sleep disorders. *The Journal of Clinical Endocrinology & Metabolism*.

[21] Späth-Schwalbe E., Gofferje M., Kern W., Born J., Fehm H. L. (1991). Sleep disruption alters nocturnal ACTH and cortisol secretory patterns. *Biological Psychiatry*.

[22] Rodenbeck A., Huether G., Rüther E., Hajak G. (2002). Interactions between evening and nocturnal cortisol secretion and sleep parameters in patients with severe chronic primary insomnia. *Neuroscience Letters*.

[23] D’Aurea C., Poyares D., Piovezan R. D., Passos G., Tufik S., Mello M. T. (2015). Objective short sleep duration is associated with the activity of the hypothalamic-pituitary-adrenal axis in insomnia. *Arquivos De Neuro-Psiquiatria*.

[24] Anstrom K. K., Miczek K. A., Budygin E. A. (2009). Increased phasic dopamine signaling in the mesolimbic pathway during social defeat in rats. *Neuroscience*.

[25] Eban-Rothschild A., Rothschild G., Giardino W. J., Jones J. R., de Lecea L. (2016). VTA dopaminergic neurons regulate ethologically relevant sleep-wake behaviors. *Nature Neuroscience*.

[26] Oishi Y., Lazarus M. (2017). The control of sleep and wakefulness by mesolimbic dopamine systems. *Neuroscience Research*.

[27] Herrera-Solis A., Herrera-Morales W., Nunez-Jaramillo L., Arias-Carrion O. (2017). Dopaminergic modulation of sleep-wake states. *CNS & Neurological Disorders-Drug Targets*.

[28] Belda X., Armario A. (2009). Dopamine D1 and D2 dopamine receptors regulate immobilization stress-induced activation of the hypothalamus-pituitary-adrenal axis. *Psychopharmacology*.

[29] Schroeder S., Burnis J., Denton A., Krasnow A., Raghu T. S., Mathis K. (2017). Effectiveness of acupuncture therapy on stress in a large urban college population. *Journal of Acupuncture and Meridian Studies*.

[30] Oh J.-Y., Kim Y.-K., Kim S.-N. (2018). Acupuncture modulates stress response by the mTOR signaling pathway in a rat post-traumatic stress disorder model. *Scientific Reports*.

[31] Bassetto R. M., Wscieklica T., Pouza K. C. P. (2017). Effects of electroacupuncture on stress and anxiety-related responses in rats. *Anais da Academia Brasileira de Ciências*.

[32] Zhao Y., Cui C., Yu X. (2017). Electroacupuncture ameliorates abnormal defaecation and regulates corticotrophin-releasing factor in a rat model of stress. *Acupuncture in Medicine*.

[33] Dias M., Vellarde G. C., Olej B., Teófilo Salgado A. E., de Barros Rezende I. (2014). Effects of electroacupuncture on stress-related symptoms in medical students: a randomised placebo-controlled study. *Acupuncture in Medicine*.

[34] Eshkevari L., Mulroney S. E., Egan R., Lao L. (2015). Effects of acupuncture, RU-486 on the hypothalamic-pituitary-adrenal axis in chronically stressed adult male rats. *Endocrinology*.

[35] Le J.-J., Yi T., Qi L., Li J., Shao L., Dong J.-C. (2016). Electroacupuncture regulate hypothalamic-pituitary-adrenal axis and enhance hippocampal serotonin system in a rat model of depression. *Neuroscience Letters*.

[36] Yano T., Kato B., Fukuda F. (2004). Alterations in the function of cerebral dopaminergic and serotonergic systems following electroacupuncture and moxibustion applications: possible correlates with their antistress and psychosomatic actions. *Neurochemical Research*.

[37] Xie C., Li L., Gao X. L., Yang W. J., Chen Y. F. (2018). Preconditionging effect of electro-acupuncture on sleep-wake in rats with insomnia due to cage change. *Journal of Clinical Acupuncture and Moxibustion*.

[38] Cano G., Mochizuki T., Saper C. B. (2008). Neural circuitry of stress-induced insomnia in rats. *Journal of Neuroscience*.

[39] McKenna J. T., Gamble M. C., Anderson-Chernishof M. B., Shah S. R., McCoy J. G., Strecker R. E. (2019). A rodent cage change insomnia model disrupts memory consolidation. *Journal of Sleep Research*.

[40] Ongini E., Bonizzoni E., Ferri N., Milani S., Trampus M. (1993). Differential effects of dopamine D-1 and D-2 receptor antagonist antipsychotics on sleep-wake patterns in the rat. *The Journal of Pharmacology and Experimental Therapeutics*.

[41] Monti J. M., Fernández M., Jantos H. (1990). Sleep during acute dopamine D1 agonist SKF 38393 or D1 antagonist SCH 23390 administration in rats. *Neuropsychopharmacology: Official Publication of the American College of Neuropsychopharmacology*.

[42] Dzirasa K., Ribeiro S., Costa R. (2006). Dopaminergic control of sleep-wake states. *Journal of Neuroscience*.

[43] Holst S. C., Bersagliere A., Bachmann V., Berger W., Achermann P., Landolt H.-P. (2014). Dopaminergic role in regulating neurophysiological markers of sleep homeostasis in humans. *Journal of Neuroscience*.

[44] Yu J., Coirini H. c., Källström L., Wiesel F.-A., Johnson A. E. (1998). Differential modulation of dopamine D1-receptor binding and mRNA expression in the basal ganglia by the D1-receptor antagonist, SCH-23390. *Synapse*.

[45] Monti J. M., Monti D. (2007). The involvement of dopamine in the modulation of sleep and waking. *Sleep Medicine Reviews*.

[46] Monti J. M., Jantos H. (2018). The effects of local microinjection of selective dopamine D1 and D2 receptor agonists and antagonists into the dorsal raphe nucleus on sleep and wakefulness in the rat. *Behavioural Brain Research*.

[47] Xu Q., Xu X.-H., Qu W.-M., Lazarus M., Urade Y., Huang Z.-L. (2014). A mouse model mimicking human first night effect for the evaluation of hypnotics. *Pharmacology Biochemistry and Behavior*.

[48] Bassett S. M., Lupis S. B., Gianferante D., Rohleder N., Wolf J. M. (2015). Sleep quality but not sleep quantity effects on cortisol responses to acute psychosocial stress. *Stress*.

[49] Lu J., Shao R.-H., Jin S.-Y., Hu L., Tu Y., Guo J.-Y. (2017). Acupuncture ameliorates inflammatory response in a chronic unpredictable stress rat model of depression. *Brain Research Bulletin*.

[50] Engel C. C., Cordova E. H., Benedek D. M. (2014). Randomized effectiveness trial of a brief course of acupuncture for posttraumatic stress disorder. *Medical Care*.

[51] Yao H., Wei D., Cai D. (2016). Effects of acupuncture on ANP and CNP in adrenal gland and CORT in plasma in rats with chronic emotional stress anxiety. *Zhongguo Zhen Jiu*.

[52] Zhou Q. Z., Pen X. H., Wu Q. F. (2008). Effect of electroacupuncture on the expression of corticotropin releasing hormone (CRH) and CRHR 1 mRNA in paraventricular nucleus of hypothalamus in stress-induced anxiety rats. *Zhen Ci Yan Jiu*.

[53] Wu Q. W. (2014). *Study on the Effect of Electroacupuncture at Shenmen and Sanyinjiao on HPA Axis Function in Psychological Stress Insomnia Rats*.

